# In Vitro and In Vivo Modeling of Normal and Leukemic Bone Marrow Niches: Cellular Senescence Contribution to Leukemia Induction and Progression

**DOI:** 10.3390/ijms23137350

**Published:** 2022-07-01

**Authors:** Myriam Janeth Salazar-Terreros, Jean-Paul Vernot

**Affiliations:** 1 Grupo de Investigación Fisiología Celular y Molecular, Facultad de Medicina, Universidad Nacional de Colombia, Bogota 111321, Colombia; mjsalazart@unal.edu.co; 2 Instituto de Investigaciones Biomédicas, Facultad de Medicina, Universidad Nacional de Colombia, Bogota 111321, Colombia

**Keywords:** leukemic niche, MSC senescence, leukemia, in vitro models, in vivo models, aging

## Abstract

Cellular senescence is recognized as a dynamic process in which cells evolve and adapt in a context dependent manner; consequently, senescent cells can exert both beneficial and deleterious effects on their surroundings. Specifically, senescent mesenchymal stromal cells (MSC) in the bone marrow (BM) have been linked to the generation of a supporting microenvironment that enhances malignant cell survival. However, the study of MSC’s senescence role in leukemia development has been straitened not only by the availability of suitable models that faithfully reflect the structural complexity and biological diversity of the events triggered in the BM, but also by the lack of a universal, standardized method to measure senescence. Despite these constraints, two- and three dimensional in vitro models have been continuously improved in terms of cell culture techniques, support materials and analysis methods; in addition, research on animal models tends to focus on the development of techniques that allow tracking leukemic and senescent cells in the living organism, as well as to modify the available mice strains to generate individuals that mimic human BM characteristics. Here, we present the main advances in leukemic niche modeling, discussing advantages and limitations of the different systems, focusing on the contribution of senescent MSC to leukemia progression.

## 1. Introduction

Since Schofield drew up his theory about the existence of a physical hematopoietic niche that drives the hematopoietic stem cell (HSC) fate [1], the possibility of some kind of fostering between niche components and cancer development has been envisaged. The HSC niche is currently described as an intricate and interconnected system composed of a large number of cell types that supply a plethora of biochemical signals essential for the production, regulation and maintenance of blood components [2,3,4]. The cellular composition and properties of the niche varies between endosteal (peripheral), and vascular (medullar) locations in the bone marrow (BM), both differentially controlling HSC functions, i.e., maintaining the HSC and progenitors’ pool, preserving quiescence, regulating differentiation and supporting regeneration during homeostasis and stress hematopoiesis [5].

In general terms, the available data indicate that alterations in the BM microenvironment can modify the signals received by stroma cells, which, in turn, can induce novel signals that support the expansion of altered hematopoietic cell subsets that eventually become more frequent and damaging [6,7,8,9,10,11,12,13,14,15,16]. The nature of those signals and the exact contribution of each component in the modification of the BM microenvironment are still unclear. What is known, however, is that the microenvironment characteristics are highly dependent on the dynamics of the niche cell populations (turnover kinetics, physiological state, mutation accumulation rate, among others) [17]. On the other hand, the cross-talk between the niche components implies that an abnormal cell population may also redesign the microenvironment in its favor [14,17,18]; for instance, the experimental evidence highlights the role of non-hematopoietic BM stromal elements in leukemia initiation and progression [19,20,21,22].

In the BM, mesenchymal stromal cells (MSC) are major and relevant components of the HSC niche, with some of them retaining their colony-forming potential and self-renewal properties; they also have the ability to differentiate into osteogenic, condrogenic and adipogenic lineages. In addition to their proliferative and differentiation potential, MSC are able to modulate neighboring cells behavior through direct contact and paracrine and immunomodulatory signaling [23,24,25,26,27,28,29,30]. However, MSC proliferation capacity is limited and during aging, the phenotype and functions of these cells change (reviewed by [31]), leading to a senescent state characterized by increased oxidative stress, dysfunction of regulatory factors, impairment of migratory and homing abilities, metabolic dysregulation and changes in their secretome composition. These senescent features have been related to decreased MSC immunomodulatory functions [32] and with the promotion of proliferation or migration of leukemic cells [18,32].

Leukemic stem cells (LSC) share several biological features with HSC, but mainly differ in the impaired regulation of signaling pathways controlling proliferation, survival and invasion [33]; they are considered a major cause of relapse in acute myeloid leukemia (AML) [34]. LSC are characterized by their ability to engraft immunocompromised mice, and the frequency of leukemic clones has been associated with clinical outcome [35]. LSC expansion is favored at the expense of normal hematopoiesis in the BM leukemic niche, where the proliferation and differentiation capacity of MSC is also eased [36,37,38].

Niche factors, such as osteopontin, CXCL12 and cytokines secreted by MSC, osteoblasts and endothelial cells may induce migration of LSC, activate signaling pathways, promote cell proliferation, and inhibit apoptosis. Niche factors can also activate LSC intrinsic self-renewal pathways, enhancing LSC survival as it occurs in AML [39] or predispose pre-leukemic cells to transformation by increasing DNA damage, as has been suggested for acute lymphocytic leukemia (ALL) [40,41]. Moreover, expression of these niche factors is modulated by the functional state of the cells in the BM microenvironment (i.e., developmental stage, inflammation, redox balance, metabolic and signaling pathways, senescence, among others).

Interestingly, healthy MSC co-cultured with B cell acute lymphoblastic leukemia (B-ALL) cells show senescent hallmarks that are similar to cultured MSC isolated from leukemic patients [30]. Moreover, several studies have shown the key role that senescent stromal cells have on remodeling the normal hematopoietic niche into a pro-leukemic one, as well as on chemotherapy resistance, relapse and residual disease in different leukemic models [18,30,42,43,44,45,46,47,48]. Cellular senescence is defined as the irreversible arrest of normal cell proliferation [49] or the stable arrest of diploid, normal cell proliferation and the cell inability to express genes required for proliferation, even in a pro-mitogenic environment [50]. Recently, cell senescence has been considered as a much more dynamic process in which senescent cells properties change, evolve and adapt in a context dependent manner (reviewed by [51,52]), with differential effects on their surroundings. In addition, tumor cells can be induced to a senescent state as a result of chemotherapy treatments; interestingly, some of these cells are able to escape senescence, become more aggressive, have a greater capacity to boost tumor growth than before the treatment, and also acquire features of cancer stem cells (gene expression patterns, signaling pathways activation and cytokine profiles), which provides experimental evidence that supports the role of senescence as a factor in cellular reprogramming and plasticity [53,54,55,56].

Senescence is induced by several types of stress (telomere attrition or DNA damage, inflammation, oxidative stress, mitochondrial malfunction and oncogenic stress) that trigger the activation of p16INK4A/Rb or p53/p21CIP1 signaling pathways. Phenotypically, it is characterized by changes in chromatin organization and gene expression that dramatically modify metabolic and secretory features in cells [57], resulting in a proinflammatory cytokine profile known as the senescence associated secretory phenotype, or SASP (reviewed by [58]). Normally, senescence works basically as a mechanism for tumor suppression and wound healing; however, it also can contribute to malignant hematological disorders by favoring the survival of LSC in the BM (reviewed by [59,60]).

In order to study these phenomena, and because of the dual role of the niche in regulating normal and malignant hematopoiesis, a rigorous assessment of the BM cell population diversity and the molecular mechanisms of MSC senescence are required to enable assays where alterations in niche cells can be experimentally tuned, and the consequences on HSC maintenance and leukemic stem cell (LSC) development can be determined. However, the understanding about cells and factors controlling human normal and malignant hematopoiesis in the BM remains limited, due the difficulty of modeling cellular relationships in the human system. Because of this, the knowledge about HSC biology and the contribution of altered MSC to cancer progression is mainly based on in vitro and in vivo murine models that have been extensively used in order to examine the physiology and relationships among BM niche cells and their role on HSC and LSC expansion [5]. In this context, but on a minor scale, other animal models have also been used [61,62,63,64]. {Fang, 2018 #184; Klinkhammer, 2014 #260; Lee, 2016 #53; Pan, 2019 #67}.

In this review, we present recent advances in leukemic niche modeling, discussing advantages and limitations of in vivo and in vitro systems. We focus on the contribution of senescent MSC to leukemia progression in these models. Understanding the role of senescent MSC in leukemia development may be useful to identify new therapeutic strategies to limit malignant cell survival and reduce treatment resistance, even by remodeling the surroundings toward a pro-normal microenvironment that is able to neutralize the leukemic niche influence and to restore HSC functioning.

## 2. Two-Dimensional and Three-Dimensional In Vitro Models

**Figure 1:**
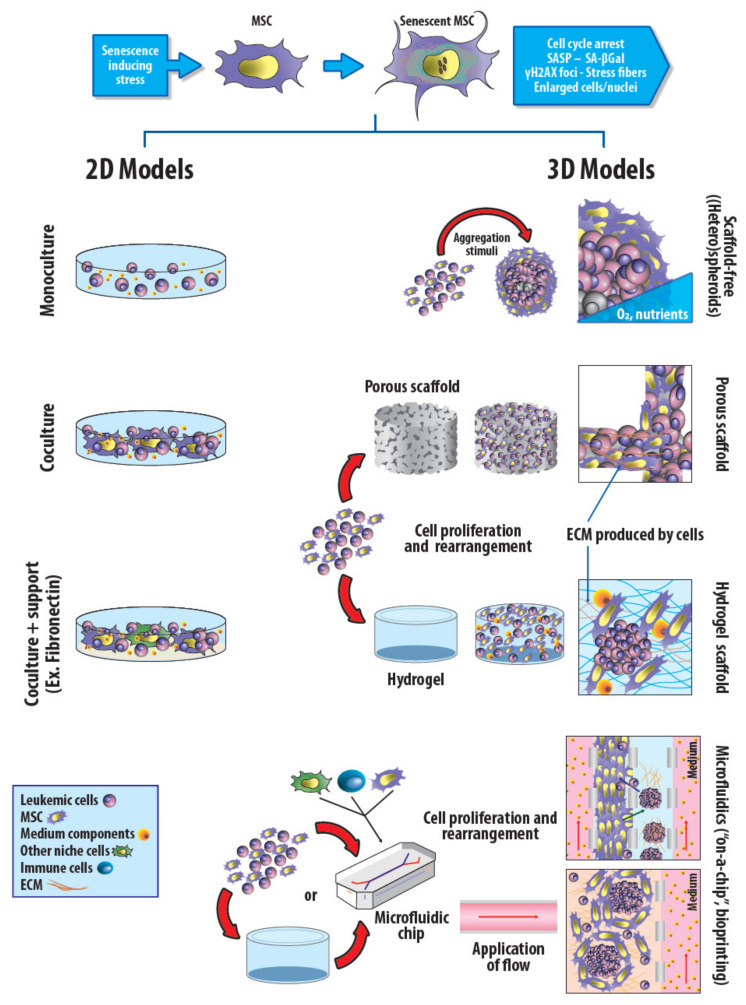
Common 2D and 3D in vitro culture models for cancer research. The use of young or senescent MSC as a support in 2D or 3D cultures for studying BM niche biology could exert differential effects on leukemic cell biology. In 2D cultures, cells grow as a suspension or as a monolayer on a flat plastic surface that can be coated or not with matrix components (fibronectin, collagen, vitronectinetc.); these kinds of systems may include one, two or more types of cells. On the other hand, 3D cultures have been developed to improve the representation of architectural, multicomponent complexity of the biochemical interactions in cancer and BM biology. Probably, the simplest way to obtain a 3D conformation is to induce the formation of spheroids composed by one or more cellular types; this can be accomplished by spontaneous formation on low-attachment surfaces, magnetic levitation, spinner flasks, among others. The use of different types of scaffolds provides a support for cell adhesion, proliferation, differentiation and migration. The scaffolds can be built with biomaterials, such as decellularized native tissues, ceramic (hydroxyapatite, bioglass), natural (collagen, fibrin, alginate, chitosan) or synthetic polymers (polyethylene glycol, polycaprolactone, poly(hydroxyethylmethacrylate), poly(lactic-co-glycolic acid)). Among the most frequently used polymers, hydrogels are preferred for their similarities to ECM mechanical properties. The application of 3D bioprinting techniques (not depicted) and the development of microchips compatible to single-cell methodologies have allowed the engineering of devices where a flow can be applied to improve cell proliferation/migration, add immune cells or test compounds in situ in a way that maintains microenvironments physically and biochemically differentiated but allow communication among them. Depending on the microchip device design and the inclusion or not of scaffolds, different culture models can be obtained. Based on [65,66,67] {Borghesan, 2020 #585; Cucchi, 2020 #586; Rodrigues, 2021 #584}.

The design of accurate in vitro models is a compulsory tool for basic research; the in vitro studies enable comparisons among different cell types, donors or donor´s ages; they also have the advantage of allowing direct cell manipulation, controlling most experimental variables, studying isolated responses and facilitating quantitative analysis. These models for cancer research vary in complexity, ranging from tumor-derived cell lines to reconstruction of 3D models of the tumor microenvironment (Figure 1).

The better understanding of both HSC and niche components biology has allowed for significant advances regarding the successful HSC expansion ex vivo, in spite of the remaining questions about the mechanisms of HSC regulation in vivo. Conventional HSC culture systems are expanded on polystyrene plates in optimized culture media, whereas the long-term maintenance of LSC is still difficult, due to the difficulty of preventing blast apoptosis to stop spontaneous differentiation and to minimize the effect of inhibitory feedback signals by differentiated cells in the culture. Patient-derived and immortalized leukemic cell lines are frequently used in experimental assays; however, their characteristics do not correspond to a functionally major LSC population [2,35,68].

The addition of support cells (e.g., fibroblasts, macrophages, MSC, among others) and the variation in biophysical parameters on the substrate to conventional 2D cultures have enabled the culture and maintenance of leukemic cells; in spite of their simplicity, noteworthy advances have been achieved by using screening approaches and culture systems supplemented with cytokines and support cells under proper oxygen and metabolic conditions. Major advances in understanding leukemic cell biology, cancer development and chemoresistance have been carried out, recreating the leukemic microenvironment in 2D culture systems for B-ALL [15,16,18,30,69,70,71,72], but more frequently for AML [73,74,75,76,77,78] and chronic lymphocytic leukemia (CLL) [79,80,81,82,83]. In this regard, a leukemic cell-free leukemic niche was established by co-culturing normal MSC with fresh conditioned medium (CM) obtained from the REH cell line, showing that normal HSC cultured under these conditions lost quiescence, increased proliferative markers Ki67 and cMyc and decreased GATA2 and p53 expression. Interestingly, MSC incubated with this CM showed senescence-associated markers [15]. On the other hand, in a human in vitro AML model used for high-throughput screening of small molecules, it was described that compounds that suppress the aryl-hydrocarbon receptor pathway have a key influence in preventing AML cell differentiation; those cells were cultured in free-serum media, analyzed under hypoxia, and cultured into different surfaces (plastic, glass and rodent fibroblasts support), showing similar results under all tested conditions [35]. Other approaches for controlling niche interactions in 2D cultures relies on the limitation of growth factors’ diffusion rate and the frequency of cell interactions by using a model of micropatterns of extracellular matrix (ECM) islands and embryonic stem cells [84], or the design of microfluidic devices that allow a controlled release of soluble compounds into the system [85].

The use of support cells in 2D systems is still the primary approach used to maintain HSC and LSC cultures. Several studies have shown the importance of MSC as niche supportive cells and providers of regulator factors for human HSC and LSC [86,87], as well as the capacity of LSC to regulate MSC phenotype and function [88]. Murine stromal cell lines have been used to expand primary human LSC in vitro; however, the leukemic blasts eventually lost their original phenotype in long-term culture systems. Thus, human MSC have been extensively used to provide support for the maintenance of those cultures [86]. Human MSC have been used successfully to culture leukemic cells from CLL [79], AML [86,88], and B-ALL [16,18]. For CLL, it was shown in vitro that cross-talk between leukemic cells and MSC improves leukemic cell viability, increasing the production of IL-8, CCL4, CCL11, and CXCL10 [79]. Moreover, direct contact of AML-BM samples with the human stroma cell line HS-5 increased proliferation, viability, and colony formation of primary AML cells in comparison with cultures without stromal support [89]. It has been also shown that LSC expressing focal adhesion kinase (FAK) were able to induce a senescent phenotype and defects in clonogenicity on MSC from healthy donors and that contact between cells was mandatory to achieve this effect [88]. A high-throughput system capable of supporting primary cells mimicking BM stroma was also developed in order to screen compounds for targeting mouse LSC in AML and differences in the effect of the compounds were found between stroma and stroma-free cultures [90]. For human ALL, it has been shown that control mechanisms exerted by normal MSC over HSC progenitors are lost in an induced leukemic microenvironment, where HSC progenitors showed altered differentiation and loss of clonogenic capacity, in spite of a higher adherence to MSC [16]. More recently, it was shown that leukemia-driven stress is able to modify healthy MSC stemness, inducing a senescent phenotype, and that MSC passage could affect the susceptibility of B-ALL to dexamethasone; however, upon withdrawal of leukemic stimulus, MSC are able to re-enter the cell cycle and recover some of their basal features [30].

MSC are rare within the BM aspirate; therefore, they must be expanded in vitro before they can be used for research or therapeutic purposes and it is clear that culture conditions and metabolic cell state can critically affect their role in supporting leukemic cells. For example, it has been shown that tension or elasticity changes in the culture substrate can rule MSC differentiation fate [91,92]. It was also suggested that dynamics of the F-actin network and early events of cytoskeleton reorganization in MSC could act as sensitive markers of lineage differentiation; the authors showed that, in contrast with the osteogenic or basal culture conditions, the actin turnover was rapidly reduced after adipogenic (few minutes) and chondrogenic (4 h) induction [93]. Meanwhile, in spite of a clear in vivo demonstration that aged animals transplanted with AML-ETO+ fostered the proliferation of LSC in BM, researchers failed in replicating the results on a short-term 2D co-culture system of pre-LSC and endosteal cells, [94], probably because of the stroma isolation method or the short-term co-culture approach used. In another study, it was suggested that MSC from AML human samples with differential expression of FAK may contribute to different microenvironmental entities mediated by differential signaling of Wnt pathway, with FAK+ cells favoring CXCL12 abundant reticular cells (CAR) proliferation and overexpression of angiogenic cytokines [88]. In the same context, the BRD7116 compound has been pinpointed as a possible therapeutic alternative for AML; its selectivity toward killing leukemic cells by affecting stroma cells was discovered using an in vitro high-throughput screening system with MSC as the supportive cells [90].

Additionally, most of the protocols add fetal bovine serum (FBS) as a media supplement for MSC, but its use has some disadvantages, such as the induction of side immunological effects, possible microbial exposure and batch variability. A study with the aim of evaluating the differences on the onset of replicative senescence between MSC cultured with FBS or human autologous serum showed that the serum source was a determinant for the time until reaching senescence and the proportion of cells in S-phase during early passages [95].

Senescence-related features in stroma cells appear to be frequently associated with conditions favoring LSC expansion in BM niche models, and several studies have addressed the question of the influence of an aged or senescent microenvironment on disease progression [29,30,96,97,98,99]. Regarding cancer progression and senescence, co-cultures of human primary AML with human BM derived-MSC with knockdown of the p16 gene reduced AML cell proliferation; moreover, co-culture of AML cells with non-silenced MSC for 6 days reduced lamin B1 expression, increased IL-6 and IL-8 production and the frequency of MSC positive for senescence-associated β-galactosidase (SA-β-Gal) staining, and induced MSC expression of p16 and p21 [42], showing that LSC can also influence the microenvironment to induce senescence compatible features that, eventually, foster their own expansion. A study using cultured multiple myeloma BM-MSC showed that these cells have a senescent phenotype (increased SA-β-Gal activity, reduced cell proliferation and upregulation of angiogenic and inflammatory cytokines), reduced both osteogenic differentiation and immunomodulatory activity and increased hematopoietic support capacity [96]. Furthermore, MSC isolated from children with malignancies (astrocytoma or myeloma) or healthy adults showed their ability to maintain B-lineage ALL cells and prevent apoptosis in vitro in a serum-free culture, but murine and stromal immortalized cell lines (M2-10B4, W18Va2 and KM-102) failed in providing such support [100]. In the same way, pediatric-derived normal MSC co-cultured with REH cells or B-ALL primary leukemic cells showed production of ROS, cell cycle arrest and increase in senescence markers (p53 gene expression and SA-β-Gal activity) [18]. Another study showed that primary ALL cells can be successfully expanded ex vivo over prolonged periods using MSC as feeder cells, maintaining self-renewal potential, clonal diversity, engraftment capacity and enabling chemotherapeutic challenges by using these long-term cultured cells for mice inoculations, in vitro clone barcoding approach and bioluminescence-labeled ALL cells for drug response assays; however, early passages of cultured MSC were the most efficient to support ALL cell growth [101].

In spite of their great utility, 2D culture systems show some constraints that limit their use to accurately reflect in vivo conditions such as the reduced availability of oxygen, the non-continuous nutrients’ access, the mechanical forces exerted by the liquid components [102], the spatial organization, and the complexity of interactions among ECM components, leukemic and stromal cells. Comparisons between both 2D and 3D systems under the same experimental conditions have been made using bone-derived materials as scaffolds to adhere patient CML-derived MSC cells supporting leukemic cells; the Ph+ subpopulation was reduced during culture in both systems, but in 3D cultures, this reduction was slower and leukemic cells showed higher CFU rates and frequency of long-term culture-initiating cells after 2 or 5 weeks [34]. Moreover, 3D models are preferred in many cases due to their capability to support complex volumetric structures with minimal toxicity, favoring cell-biomaterial interactions and nutrient exchange [103].

Hence, several protocols for bioengineering 3D HSC niches have been established for mouse and human HSC and LSC expansion [4,103,104,105,106,107,108,109,110,111]. A well-designed artificial niche must be adaptable and scalable, facilitate selective incorporation of a variety of biochemical signals and allow the use of high-throughput or single-cell techniques, in addition to overcoming biomechanical and biotransport restrictions [68]. A big step towards the modeling of the leukemic niche was the possibility to grow leukemic cells as spheroids [107,112,113], or their implantation into polymer matrixes [37,109,112] or inside porous scaffolds [103,105,111,114]. The use of bioreactors or microfluidic devices further improved nutrient supply and increased the number of parameters involved on the niche building [115,116,117,118,119]. Much of the current research relies on achieving a more detailed niche model by combining different substrates with varied stiffness, soluble factors, more cell types and a vascular system integrated in a single model [120] and also using methodologies that allow single-cell fate tracking.

Inorganic (hydroxyapatite, tricalcium phosphate), natural (collagen, fibrin, heparin) or synthetic polymers (polyurethane, poly-L-lactic acid, polyethylene glycol) have been tested as scaffolds to provide a 3D structure for normal and leukemic niches (reviewed by [120]); polymeric biomaterials substrates (polyethylene terephthalate, tissue culture polystyrene and polyether sulfone) are more adequate because of their defined composition, surface chemistry and toxicity profile. Open-cell foam scaffolds made with low-density biomaterials with distinct levels of elasticity were also adopted as analogs of the trabecular bone and used with stromal cells to support HSC expansion [2,85]. Other 3D models rely on woodpile structures fabricated by two-photon polymerization of different photosensitive polymers or hydrogels; these scaffolds are adequate to study mechanical properties on the adhesion and proliferation of MSC [103]. Indeed, scaffold type, matrix stiffness, and architecture applied to HSC or LSC cultures differ greatly and, consequently, can dramatically influence cell fate, as described for MSC [91,92].

Improvements to 3D culture systems were developed in order to capture and perform single-cell analysis on HSC cells maintained in microfluidic devices [117], or to compare the effect of scaffold geometry on human MSC growth and differentiation by combining low intensity pulsed ultrasound and 3D printing techniques [114]. Another innovative approach was the design of a biomimetic microdevice by engineering new bone in vivo and perfusing it with culture medium in a microfluidic system; this model enabled the culture of living bone with a hematopoietic cell composition that resembles the natural BM environment and supports HSC in similar proportions to in vivo models, maintaining their spatial positions within a 3D niche in vitro [119]. Using a system based on a polyethylene glycol hydrogel, a peptide anchor and a MSC coculture integrated to a bioreactor that allowed the perfusion with medium while culturing HSC, it was possible to determine the variations regarding differentiation, drug resistance and maintenance of stemness for HSC progenitors between static and dynamic 3D cultures. However, cell proliferation was not influenced by the culture method [102]. Using confocal microscopy and RT-PCR over a microvascular, endothelialized platform fabricated in a type I collagen matrix and designed to evaluate multicellular interactions and cell trafficking, it was revealed that HSC progenitors and AML cells showed different adhesion rates depending on the type of feeder cells (primary MSC or fibroblast cell lines HS27 and HS5) [118].

Much of the research regarding leukemic niche modeling is related to the screening of therapeutic compound targeting of LSC in the BM [121]. Using a synthetic polyglycolic acid/poly L-lactic acid (PGA/PLLA) 90/10 copolymer scaffold and primary MSC as co-cultured cells, it was shown that resistance to doxorubicin and cytarabine for three leukemic cell lines (HL-60, Kasumi-1, and MV411) was greater when leukemic cells were cultivated in 3D systems [122]. Similar results were obtained using a poly-ethylene glycol and heparin hydrogel 3D system functionalized with a peptidic ligand for adhesion, angiogenic factors and co-cultured with HUVEC and MSC; in this system, four leukemic cell lines (KG1a, MOLM13, MV4-11 and OCI-AML3) and patient-derived AML cells showed lower sensibility to daunorubicin and cytarabine compared with 2D suspension cultures [112]. For the ALL model, a tri-cellular (SUP-B15 cell line, MSC and osteoblasts) microfluidic platform based on a polydimethylsiloxane resin was designed to evaluate the response of leukemic cells to cytarabine in mono or co-culture comparing 2D and 3D systems, reinforcing the findings of higher resistance to chemotherapeutic in 3D cultures. In addition, the researchers highlighted the occurrence of discernible niches on their 3D model [115]. More recently, an improved chip 3D model was designed to mimic different BM regions (central venous sinus, medullary cavity and endosteum), enabling spatial separation, intercellular communication, cytokine modulation, and real time and live-cell imaging; this 3D model resembled in vivo BM tissue in terms of structure and cell composition. The researchers confirmed that MSC and osteoblasts seemingly induce leukemia dormancy via osteopontin signaling, and more importantly, the system compatibility with single-cell technologies allowed the generation of a more detailed map of interactions among different types of B-ALL cells, samples and microenvironments inside the artificial BM model and how these interactions affect the sensitivity of B-ALL to chemotherapeutics [123].

In spite of the advances on 2D and 3D in vitro models, they still have physiological constraints because they failed to capture the whole complexity of the tumor microenvironment; their biggest limitation relies on the lack of correspondence with a whole organism. This limits the use of the in vitro models for some experimental approaches (e.g., cell-drug interactions and engraftment assays, among others), making in vivo studies in animal models necessary.

## 3. Animal Models

Research on human subjects is strongly limited by ethical, technical and legal issues; therefore, in most cases, clinical trials on people are preceded by studies on non-human species. A broad variety of animals, including mammals and non-mammal species, have been used for studying leukemic development, senescence and aging processes; this large spectrum is valuable because it helps one to discover the generic molecular mechanisms that govern those processes. However, many features may be unique for a particular class of animals. For example, the finding that the target of rapamycin (mTOR) pathway plays a key role on vertebrate senescence was previously discovered in yeast, nematodes and flies [124]; however, human and naked mole-rats have a far lower incidence of cancer than mice at any age and some mouse strains (e.g., AKR/J) are highly susceptible to neoplasia [125]. In spite of the huge advances regarding transgenic manipulation or xenotransplantation of mice, this kind of technology is rarely used in other small rodents or other animal models. Fishes, birds, rabbits, dogs, cats and non-human primates (mainly rhesus macaques *Macaca mulatta*) have been used, but on a considerably lesser scale than mice and rats (reviewed by [126,127,128]). Currently, there is general awareness that no single model can reflect completely the complexity of human cancer and senescence mechanisms, but most studies still rely on a limited range of models.

### In Vivo Rodent Models

Rodents are the organisms that better fulfill the requirements of a desirable animal model (life table data available, short life span, controllable environmental and pathological conditions for maintenance, well-studied genetics and feasible cost). Besides several mice strains that are discussed in more detail below, other small rodents, such as rats (*Rattus norvegicus*, mainly Fischer 344, Sprague-Dawley, Wistar, and Long-Evans strains), Syrian hamster (*Mesocricetus auratus*), the naked mole rat (*Heterocephalus glaber*), and the Mongolian gerbil (*Meriones unguicultatus*), have been used as models for aging and senescence studies (reviewed by [126,128]).

**Figure 2:**
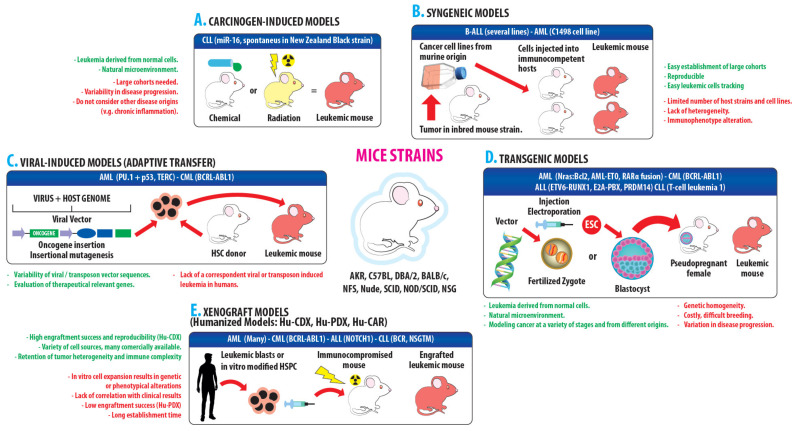
Approaches for developing leukemia murine models. (**A**) Carcinogen-induced models: leukemic mice induced by carcinogen administration (chemical, ionizing radiation). (**B**) Syngeneic models: leukemic mice established by injection of murine cancer cell lines in immunocompetent hosts. (**C**) Mosaic, viral, and transposon induced models: leukemic mice originated from oncogenes engineered in a viral vector through transfected murine HSC, causing oncogene or aberrant proto-oncogene expression or disruption of tumor suppressor genes; similar results are obtained from transposon sequences inserted into the host genome. HSC can be manipulated using either retroviral transduction or genome editing techniques. (**D**) Transgenic models: leukemic mice established by genetic manipulation of cancer-causing genes injected or electroporated into embryonic stem cell (ESC) or fertilized zygotes; ESC that have incorporated the vector are injected into tetraploid blastocysts. These blastocysts or the transformed zygotes are transplanted into pseudopregnant receptive females, whose offspring is monitored to detect leukemic individuals. (**E**) Xenograft models: leukemic mice are obtained from immunocompromised hosts that have been inoculated with primary patient samples or human cell lines. Hu = Mice reconstituted with human immune system. Hu-CDX (cell line xenograft) originated from immunodeficient mice inoculated with human cancer cell lines. Hu-PDX (human patient-derived xenograft) originated from patient’s biopsies without applying ex vivo culturing prior to transplantation. Hu-CAR: immunocompetent mice inoculated bearing human tumor xenograft and administered a CAR therapeutic. Green letters: advantages. Red letters: disadvantages. Blue inset: examples of gene targets for each type of leukemia studied with the model. The graph is based on [129], with additional information from [145,147,148,149].

Leukemic mice models are generated by exposure to carcinogenic chemicals, genomic integration of oncogenes delivered by virus or transposons, implantation of modified embryos and zygotes transplanted with ex vivo manipulated HSC (transgenic models) or injection of primary patient samples or human cell lines into immunocompromised host animals (xenograft models) (Figure 2) (reviewed by [129]). Much of the scientific advancement in biomedical research has been obtained from studies in transgenic mice [130]; however, several components of the mouse genetic background or the immune system do not faithfully translate to human biology [131,132,133]. Some gene functions can be different among species [134] and many drugs and pathogens are species-specific [135]. Even though animal models are valuable tools for research, the expenditure is high, drug screening requires a large amount of animals and, biologically, model animals cannot completely mimic human BM microenvironments. These issues emphasize the need for better strategies to improve human systems modeling in vivo; in this context, new results and extensive reviews regarding animal models for leukemic research are periodically published [127,129,136,137,138,139,140,141,142,143,144,145,146].

Xenograft mice models arose after the development of immunodeficient mice bearing the IL-2 receptor common gamma chain (*IL2rγ^null^*) and the combination with mutations in the protein kinase DNA-activated catalytic polypeptide gene (*Prkdc^scid^* or *scid*) or in the recombination activating gene 1 or 2 (*Rag1^null^*, *Rag2^null^*) that allowed the generation of mice lacking adaptative immunity and exhibiting deficiencies of innate immunity. Immunodeficient *IL2rγ^null^* mice have enabled the successful engraftment of various human cancers; the strains NSG (NOD.*CgPrkdc^scid^Il2rγ^tm1Wjl^*), NOD (NOD.*Cg-Prkdc^scid^Il2rγ^tm1Sug^*) and BRG (C; 129S4-*Rag2^tm1Flv^Il2rγ^tm1Flv^*) are the three kinds of immunodeficient *IL2rγ^null^* mice more frequently used as in vivo platforms for identifying and testing drug targets, for the identification of LSC and investigation of metastasis, as well as preclinical models for the evaluation of new therapeutics [141,146]. Humanized mice models, in which the human immune system is partially reconstituted in mice by direct genetic manipulation or by engrafting HSC or stromal components of human origin into the BM of immunosupressed animals, have been developed from these strains [150]. The development of mice strains showing human-like immune system characteristics improves engraftment rate success, reflects the changes that can occur in human patients over time and allows a more precise evaluation of the preclinical efficacy and safety of immunotherapy [151]. In another approach, syngeneic models were developed using murine cell lines or virally-transduced murine HSC to express target genes that result in leukemia initiation. These models do not need a complex breeding process as the genetically modified mice strains do, they allow the study of the disease in immunocompetent hosts, and have become the most commonly used preclinical models for immunotherapy evaluation; however, syngeneic mice lack genomic and microenvironmental heterogeneity because of the limited number of cell lines that can be implanted in a restricted number of inbred strains of mice [148,152,153]. Recently, by using lentiviral vectors for simultaneously transducing an oncogenic mix into immunocompetent animals, it was possible to establish AML-like and CLL-like leukemic lines using syngeneic mice [154].

One of the major limitations of transgenic and xenograft models is that, in spite of the possibility of genetically manipulating and transferring human HSC into immunodeficient mice, it is not possible to also transplant the entire human BM niche into the animal recipient; in consequence, the injected human cells deal with a murine microenvironment that does not mirror human BM interactions. Several studies have focused on improving this constraint and the engraftment success rate by using humanized mice models. For example, by using NOD/SCID/*IL2rγ^null^* mice as hosts, an in vivo extramedullary bone model was developed by subcutaneous injection of a mix of human BM-derived MSC and peripheral blood-derived endothelial colony-forming cells cultured on a matrix containing laminin and collagen IV; the functionality of the model was tested by inoculating human MOLM13/Luc/GFP leukemia cells and evaluating the engraftment success, demonstrating that these AML cells can easily engraft into the extramedullary bones. The model also allowed the researchers to test the effect of HIF-1α silencing on MSC, showing a decrease in leukemic cell engraftment in the extramedullary bones derived from silenced MSC cells and demonstrating that HIF-1α expression is a key component of engraftment, by directly up-regulating CXCL12 expression [155]. In another study using *RAG2^null^* *IL2rγ^null^* mice as recipients, the researchers implanted biphasic calcium phosphate particles loaded with human MSC under the mice skin and evaluated the homing and differentiation of CD34^+^ cells isolated from umbilical cord blood and the ability to support the growth of patient-derived multiple myeloma cells expressing the luciferase reporter. They showed that this scaffold system functionally supported engraftment for normal and leukemic cells and lineage differentiation of HSC [156]. Both studies highlight the importance of a humanized microenvironment for studying human leukemic niches in animals; however, technical advances are still required in order to achieve an identical human immune function in recipient animals (e.g., HLA alleles matched to donor human cells, development of lymphoid architecture, identification of human-specific factors needed for optimal human cell function that are absent in mice, among others). It is also necessary to prevent the development of graft vs. host disease, which occurs in many of the human immune engrafted models, and validate the results obtained in the models described here [146,157].

On the other hand, senescence study using in vivo models has been challenged by experimental constraints, such as the difficulty to identify senescent cells in a living organism, the lack of standardized and validated in vivo biomarkers, as well as the long-term monitoring needed for studying murine aging [158]; nevertheless, several systems for the study of senescence and its role in aging and cancer development in living rodents have been developed, including the use of several rat strains to characterize stromal cell senescence by ex vivo culture techniques [62,65,159,160], and using progeroid murine lines [161] and senescence accelerated mice (SAM) strains with different life spans and senescence associated features [162,163]. These mice display atypical expression of genes encoding cell cycle checkpoints, DNA repair and nuclear proteins, ROS scavenging enzymes and signaling pathways components and are considered valid models for studying both senescence and aging. However, none of these models have been extensively used for leukemia research and most of the time, senescent cells have been only detected using end-point measures, such as qRT-PCR analysis, in situ hybridization, immunoblot or SA-β-Gal activity on blood, fresh or euthanized tissues. Alternatively, peripheral blood lymphocytes, plasma or serum from the animal are also collected to detect changes in the secretion of SASP factors [161,164,165].

More advances have been achieved when using transgenic and xenograft models for studying senescence and aging roles in hematopoietic regulation and leukemia progression in the context of the BM microenvironment. First of all, as explained for in vitro models, murine models also support the role of MSC in the development and fate of LSC and pre-leukemic cells. Using wild-type and periostin-deficient transgenic mice in a BALB/c background, the researchers revealed that BM-derived MSC expressed higher levels of periostin (via STAT3 activation) when co-cultured with B-ALL cells and that periostin deficiency in MSC decreases CCL2 expression in co-cultured B-ALL cells; CCL2 expression in these cells is regulated by the ILK/NF-kB pathway [166]. In a SCL-tTA/BCR-ABL (CD45.1/CD45.2 C57BL/6) transgenic, inducible CML mouse model, targeted deletion of CXCL12 from MSC, but not from endothelial cells, reduced normal HSC numbers, promoted LSC self-renewal and increased sensibility of LSC to tyrosine kinase inhibitors, possibly through enhanced EZH2 activity [167]. In the same context, a transgenic murine model for AML (based on MLL-AF9 fusion gene expression and DsRed reporter) was designed to obtain a high number of LSC to perform a high-throughput screening of small molecules with anti-leukemic properties in a co-culture system, with primary BM-MSC derived from actin-GFP mice or GFP-expressing BM stroma-derived OP9 cells [90].

Second, many of the physiological changes described for MSC in pro-leukemic and leukemic microenvironments are equivalent to those occurring during BM-aging and senescence [30] and it has been suggested that MSC are similarly affected by different types of leukemic cells, regardless of the particularities of each model [52]. In this context, anatomical changes in bones and blood vessels observed in both old mice and elderly humans hint at the remodeling of the entire niche microenvironment during senescence and aging, affecting HSC features (reviewed by [5,168]). The factors driving remodeling of the BM microenvironment in leukemia are not well understood; however, a proinflammatory landscape, which is a hallmark of cellular senescence, is a key factor in this process [169]. It is not clear, however, if the changes in the hematopoietic system precede the BM inflammatory phenotype or vice versa, or if the aging of the hematopoietic system could be reversed [94]. For example, using an oncogenic *Nras*-mutant mouse model, it was experimentally proved that a senescent BM microenvironment promotes a chronic myelomonocytic leukemia-like condition and that intra-BM transfusion of young MSCs can rejuvenate the senescent BM microenvironment [45]. However, the transfusion was only insufficient to suppress disease development in an MLL-AF9 AML mouse model [170].

To better investigate how stromal dysfunction can foster the development of cancer and to elucidate the cellular and molecular mechanisms of MSC senescence that contribute to leukemia progression, other approaches relying on senescent cell identification and ex vivo characterization using fluorescent tags [171], as well as detection and/or selective elimination of senescent cell populations from living organisms, have been used [158,171,172,173,174]. For instance, changes in senescence and differentiation-related genes expression associated with aging MSC were investigated using these models [175]. Specifically, the role of p16 in senescence has been studied in vivo using several transgenic mouse strains modified to express fusion proteins or probes that allow the tagging, selective depletion and single-cell analysis of p16 expressing cells [42,158,172,173,176]. In this context, the p16-3MR mouse model has been used for studying the role of senescence in breast cancer progression, chemotherapy and relapsing [176]. This model also demonstrated that AML blasts not only induce a senescence process driven by leukemia-generated NOX2-derived superoxide and regulated by p16^INK4a^ expression in stromal cells within the BM, but also that depletion of SASP and senescent stromal cells promote leukemic mice survival [42]. However, a knock-in approach using a luciferase reporter for lifelong monitoring of p16^INK4a^ in a cohort of mice showed strong induction of p16^INK4a^ in the healthy stroma of early neoplasms, but a lack of association between the expression of this senescence marker and cancer-related mortality [158]. Here, it is important to stress that care should be taken when assessing a senescent phenotype using few markers; for instance, high SA-β-Gal activity and p16^INK4A^ expression can be induced in non-senescent macrophages as part of a reversible polarization response to immune stimuli in mice, with hemizygous p16^INK4a^ knock-in of the luciferase gene reporter [177]. In addition, in contrast to what occurs with p16^INK4a^, other genes associated to senescence in mice do not have similar expression patterns in humans, as it happens with the alternative products of INK4 locus (p16 and ARF) [178] and Rb genes [134].

Reactive oxygen species (ROS) signaling clues are important to maintain quiescence and define differentiation fates of HSC and LSC [179], and also play a key role as senescence triggers; however, oxidative stress is rarely used as an induction factor in mice, except for the D-galactose model (reviewed by [165]) and in vivo ROS determination is challenging. In spite of this, mice models have been used to study human ROS responses because of the metabolism similarity between both species and the possibility of long-term follow up and evaluation of ROS effects in complex tissue microenvironments [180]. Methods for non-invasive ROS measurements have been tested for transgenic and xenograft murine models of tumors [181,182,183,184], but conditional genetic knockout mice have been used more frequently. In this respect, a transgenic mouse model with constitutive deficiency of connexin-43 (Cx43, a key structural component of gap junctions in BM) and in vitro experiments with the FBMD-1 stroma cell line demonstrated that the hematopoietic microenvironment can function as a major oxidative stress homeostasis orchestrator, facilitating ROS scavenging through their transfer from stressed HSC to stromal cells through Cx43 complexes. Moreover, surviving HSC from H-Cx43-deficient mice displayed senescence features (p16^INK4a^ up regulation, cell cycle arrest after chemotherapy and decreased regenerative capacity) and activation of p38MAPK/FOXO1, a ROS concentration-dependent signaling pathway [185]. A combined strategy of in vitro and in vivo experiments using a patient-derived AML xenograft model in NSG mice demonstrated that mitochondria transfer from stroma cells to AML blasts occurs via leukemia-derived tunneling nanotubes and that ROS levels regulate this transfer through the NOX-2 signaling pathway; mitochondria transfer exerts a pro-leukemic effect in these mice [186]. Also for AML, a doxycycline-inducible rtTA;MLL-AF9 mouse strain was used to show the important role of GSH and GPX enzymes dependent of BM-MSC Nestin^+^ as antioxidant protection for leukemic cells against chemotherapy [21]. In a study that looked for secondary drivers of leukemogenesis in B-ALL, it was reported that JAK mutations can alter the fate of leukemia clonal evolution through ROS-induced DNA damage; the researchers used a mouse model driven by PU.1/Spi-B deletion (Mb1-CreΔPB) [187].

The relevance of mice models for studying telomere-related human biology is also debated [188,189,190]. Telomere length is determined genetically and their shortening is considered one of the trademarks of senescence and aging; telomeres’ length and the rate of shortening vary among the species. Although the basic biology of telomeres is the same in mice and humans, their characteristics and functions are distinct and the alterations in telomere length regulation may result in different manifestations [189]. Humans have shorter telomeres than mice, but mice life span is brief and their rate of telomere shortening is up to 100-fold higher [191,192]; therefore, lack or dysfunctional telomerase has severe or fatal effects on humans, while murine models deficient for telomerase have been developed with diverse outcomes. For instance, a reversible murine model of telomerase deficiency regulated by tamoxifen was used to determine the influence of telomerase activity on erythroid and myeloid lineage differentiation; the F5 progeny of the reverse transcriptase (TERT) deficient mice showed shortened telomeres and a reduced HSC counting, among other pathological conditions. The reactivation of telomerase activity restored HSC and HSPC proliferation, normalized the DNA damage response and improved erythropoiesis [193]. It has also been shown that chimeric adult mice derived from embryonic stem cells with hyper-long telomeres have extended life span with improved mitochondrial and metabolic parameters, display a lower number of cells with global or telomere-induced DNA damage with aging and lower levels of the p21 senescence marker; these mice were also less prone to developing spontaneous tumors [194].

The use of mice models of oncogenesis led to the theory that telomerase deficiency operates initially as a pro-oncogenic pathway, probably through chromosomal instability but continuous inactivation of telomerase in tumor cells would decrease tumor growth [189]. So, mice with knockout of the telomerase RNA component (TERC) were used to evaluate the role of telomerase in leukemogenesis for AML and T-ALL subtypes. By comparing TERC^−/−^ individuals from the F1 and F3 generation (with differences in telomere length and telomerase activity) and the progeny of different back-crosses between them, the researchers concluded that leukemia can be induced in the absence of telomerase activity and the aging microenvironment induced by telomere dysfunction can accelerate it; however, short telomeres can prevent leukemogenesis by inducing DNA damage [195]. Conversely, other studies highlight the high activity of telomerase in cancer cells, as a mechanism that allows these cells to continually replicate despite accelerated telomere shortening and as a requirement for leukemia maintenance [196]. A previous study using a MLL-AF9 AML model also supports the conclusion that telomerase activity is not mandatory for leukemia initiation, but its deficiency negatively affects the self-renewal capacity and frequency of functional LSC and reduces the leukemia burden; LSC lacking telomerase activity showed chromosomal instability, increased apoptosis and induction of the p53 pathway [197]. Telomere deregulation and dysfunction also play important roles at specific phases of CLL progression; multiple components of the telomere system are affected and related with activation of DNA damage response and cell-cycle checkpoint deregulation (reviewed by [198]).

Another interesting approach to study biological processes in vivo, including hematopoietic, senescence and leukemic pathways, is the use of two-photon microscopy [199,200,201,202]. This technique was useful for demonstrating that HSPC from aged mice, compared to those from younger animals, showed distinct relationships to BM architectural features, decreased adhesion to the stroma and a tendency to increase myelomonocytic differentiation when transplanted into younger mice hosts [199]. A fluorescent probe engineered to be detected by two-photon microscopy [200,201] was validated for the detection of senescent cells in mice bearing melanoma tumors and treated with palbociclib to induce a senescent phenotype. The fluorescent signal was enhanced in tumors treated with the chemotherapeutic agent and the specific senescence signal emission was only observed in senescent tumors cells without the expression of Ki67 and phosphorylated Rb protein reduced [200]. Another group developed a new method by merging fluorescence two-photon microscopy with imaging-optimized two-photon microscopy of phosphorescence lifetimes (FaST-PLIM for ‘fast’ scanning two-photon phosphorescence lifetime imaging microscopy); using FaST-PLIM, intravital oxygen and cell dynamics were measured in CD11c-EYFP, B-ALL mice carrying a double reporter. In this way, they were able to measure local O_2_ availability in murine BM and found a greater frequency of hypoxic regions in advanced-stage B-ALL individuals [203].

As already described, traditional and cutting-edge technologies used for senescence assessment and BM modeling support the key role of senescent/aging BM microenvironment-related alterations in the initiation and progression of hematological malignancies. Future research could be used, not only to improve the understanding of HSC regulation by the niche or to better elucidate the molecular mechanisms that swing cells targeted by senescence clues toward apoptotic or malignant transformation, but also to modulate or to revert senescence progress at different stages, to evaluate how the SASP impacts the interaction between senescent cells and the immune system over time, and to improve current therapies against leukemias using senolytic approaches. In fact, preclinical models suggest that senolytic drugs would be able to decrease the in vivo frequency of senescent cells, reduce inflammation and improve patients welfare (reviewed by [204]). In this regard, the first generation of natural or synthetic compounds with senolytic effects, such as dasatinib, flavonoids (quercetin, fisetin) [205,206,207], Navitoclax [208,209], nutlin 3 [210], among others, have been tested alone or in combination as a chemotherapy for cancer. More recently, second generation senolytic treatments including vaccines [211] and nanoparticles [212] have been also developed in order to improve the efficacy and decrease the offside effects of senolytic-based treatments. Thus, various tools have emerged to directly and indirectly attack hematological malignancies. Developments are needed to better understand the cellular and molecular mechanisms to determine the ideal timing to provide these new treatments.

## 4. Conclusions

In recent years, there has been a growing interest regarding the role of the senescent BM microenvironment in leukemia development, but the causal relationship between both events is still unclear, partly due to the wide range of in vitro and in vivo models available for leukemia study and the variety of protocols used for senescence study. In spite of their value, as tools for basic research and pre-clinical trials for drug efficacy evaluation, there is still a need for better BM leukemic niche modeling, as well as a standardized methodology for cellular senescence assessment. In the meantime, and because different methods of inducing senescence have singular characteristics and effects, care must be taken to ensure that the chosen model is well-suited to the experiment and its objective. Specifically, it is a matter of discussion regarding how representative mouse models of hematopoietic aging or leukemia development are and if further modeling should be conducted in aged mice instead of young animals to understand the context dependency of genetic and pharmacological perturbations. This is particularly relevant when discussing murine models for pediatric leukemias, where differential effects of young vs. old mice’s BM microenvironment on B-ALL cells can be observed [213]. Concerns about animal welfare, costs, validity and applicability of outcomes when animal models are used must also be taken into consideration; however, applying the most recent biotechnology and technical advances, such as single-cell and high-throughput platforms, will allow for better exploitation of the biological resources.

## Data Availability

Not applicable.
